# Effect of Baking Temperature on the Phenolic Content and Antioxidant Activity of Black Corn *(Zea mays* L.) Bread

**DOI:** 10.3390/foods10061202

**Published:** 2021-05-26

**Authors:** Gracia Patricia Blanch, Maria Luisa Ruiz del Castillo

**Affiliations:** Instituto de Ciencia y Tecnología de Alimentos y Nutrición, Consejo Superior de Investigaciones Científicas (ICTAN-CSIC), Juan de la Cierva 3, 28006 Madrid, Spain; gblanch@ictan.csic.es

**Keywords:** *Millo corvo*, black corn, phenolics, antioxidant activity, bread, heating

## Abstract

Black corn is known for its health-promoting properties, which are due to its high content of bioactive phytonutrients. However, the high temperatures required during the processing of bakery products usually trigger thermal degradation, and therefore, the loss of all labile bioactive compounds. In the present study, we evaluated the effect of baking temperature on the phenolic content (i.e., TPC, TAC and individual phenolics) and antioxidant activity in black corn (*Millo corvo* variety) bread. As a result, baking always resulted in a general decrease in TPC, even at 150 °C. In contrast, TAC only decreased when temperatures as high as 180 °C were applied. Some relevant individual phenolics were preserved during the whole process as long as 150 °C was used. In particular, the content of the major anthocyanin, namely, cyanidin-3-*O*-glucoside, hardly decreased from the raw flour to the final bread. The loss of antioxidant activity of *Millo corvo* raw flour during bread baking was avoided by heating at 150 °C. These results demonstrate the appropriate temperature to bake *Millo corvo* corn bread without losing the antioxidant characteristics and health-promoting properties of the starting black corn.

## 1. Introduction

Corn, together with rice, is one of the most cultivated cereals in the world, and since it is gluten-free, it is also suitable for consumption by those with celiac disease. There are several varieties of corn; however, in recent years, black corn has become particularly popular. From a marker standpoint, black corn is a natural colorant with a plant origin that can be very interesting as an alternative to artificial additives. More importantly, black corn possesses excellent nutritional properties due to its high content of relevant phytonutrients with functional roles in human health [1].

In this respect, black corn contains some phenolics, carotenoids and particularly high content of anthocyanins [2]. Summing up the results of many studies, anthocyanins possess anticarcinogenic activity, cardiovascular disease prevention, obesity control and diabetes alleviation properties [3]. Among the anthocyanins reported in black corn, cyanidin-3-*O*-glucoside is the most abundant [4]. Beneficial health-related effects of non-anthocyanin phenolic compounds (e.g., quercetin, kaempferol, phenolic acids) that are present in black corn have also been extensively reported [5].

Corn-based foods require a thermal process prior to consumption. In this context, the inadequate selection of baking time and temperature may cause breakdown problems, such as cracking and checking [6], affecting the final quality [7]. The baking conditions may also bring about the degradation of thermally unstable compounds, such as anthocyanins [7]. In fact, many processed food containing anthocyanins exhibited a decrease in their content as a result of the thermal treatment [8]. The goal of this research was to evaluate the effect of baking temperature on the content of phenolic compounds in general and on anthocyanins in particular, as well as on the antioxidant activity of bread baked using black corn *(Millo corvo* variety). To this end, the phenolic composition and antioxidant properties of the final bread obtained were compared with those of the starting *Millo corvo* flour to select the heating conditions that enabled the flour antioxidant content to be preserved. To date, no bibliographic report can be found in the literature about bakery products baked using *Millo corvo* corn.

## 2. Materials and Methods

### 2.1. Chemicals

Ultrapure water was obtained from a purification system (Millipore Milford, MA, USA). Both formic acid and MeOH (HPLC grade) were obtained from VWR Inc. (Bridgeport, PA, USA). 2,2-diphenyl-2-picrylhydrazil (DPPH) and sodium carbonate standards were supplied by Sigma-Aldrich (Steinheim, Germany). An antioxidant capacity lipid-soluble (ACL) kit was acquired from Sigma-Aldrich (Steinheim, Germany). Foulin–Ciocalteu reagent was obtained from Merck (Darmstadt, Germany). Chlorogenic, caffeic and ferulic acids, as well as quercetin and quercertin-3-glucoside standards, were purchased from Sigma-Aldrich (Steinheim, Germany). Cyanidin-3-*O*-glucoside standard was provided by Extrasynthase (Genay, France).

### 2.2. Materials

For the experiments, black corn (*Zea mays* L., *Millo corvo* variety) and conventional white corn produced and supplied by Asociación Cultural Meiro (Morrazo, Pontevedra, Spain) were used. Flour samples were prepared via dekerneling corn by hand and then grinding the whole grain using a traditional stone corn mill. Flour samples were immediately stored at −14 °C until their use. The preparation of bread was accomplished as follows: dough from flour was carried out by mixing 35 g of raw flour with 10% of water. The mixture was then optimally hand kneaded to a smooth dough. The breadmaking process was performed in a scale-down method by adding 4.6 g of fresh yeast to 35 g of the dough previously prepared. After that, it was kneaded by hand up to a homogenous mixture, which was finally baked in an oven at 150 °C for 60 min. Two batches were run for each sample.

### 2.3. Extraction

For the experiments, a 5 g sample weight was used. Polyphenols were extracted by adding 60 mL of solvent methanol/acetone/water (35/35/30) to the sample. The resulting mixture was homogenised with an Ultraturrax homogeniser (IKA, Sigma-Aldrich, Madrid, Spain) for 10 min and then centrifuged at 2500 rpm at 15 °C for another 10 min. The solvent was removed and an additional 6 mL of methanol/acetone/water (35/35/30) was added to the extract, which was re-extracted. Then, the extract was taken to dryness using a rotary evaporator. Finally, the dry extract was stored at −14 °C until analysis. The extractions were carried out in triplicate and both the *Millo corvo* flour and bread samples were analyzed as explained below.

### 2.4. Total Phenol Content (TPC)

The equipment used for TPC measurements was a Beckman Coulter DU-800 spectrophotometer (Barcelona, Spain). The method applied was based on the oxidation of the hydroxyl groups of phenols in basic media by the Folin–Ciocalteu reagent, as described in the literature [9]. In brief, 0.5 mL of Folin–Ciocalteu reagent and 10 mL of a sodium carbonate solution (75 g L^−1^) were added to a 0.1 mL volume of the extract (20 mg mL^−1^). The final mixture was made up to 25 mL with distilled water. After 1 h, the absorbance was measured at 750 nm against a blank (i.e., mixture without the reagent). The results were expressed as milligrams of gallic acid equivalents (GAE) per 100 g of sample. All the analyses were carried out in triplicate.

### 2.5. Total Anthocyanin Content (TAC)

Total anthocyanin was quantified using the pH differential method reported elsewhere [10]. *Millo corvo* corn flour and bread samples were diluted with 0.025 M potassium chloride buffer solutions at pH 1 and with 0.4 M sodium acetate buffer at pH 4.5. A 400-700 nm sweep was carried out using a spectrophotometer (Beckman Coulter DU-800 spectrophotometer, Barcelona, Spain). TAC was expressed as milligrams of cyanidin-3-*O*-glucoside equivalents per 100 g of sample based on a molar extinction coefficient of 26,900 L cm^−1^ and a molecular weight of 449.4 g/L.

Total absorbance was measured using the equation:

Abs_t_ = (Abs_520 nm_ − Abs_700 nm_)_pH = 1_ − (Abs_520 nm_ − Abs_700 nm_)_pH = 4.5_

### 2.6. DPPH Activity

Antioxidant activity was determined using a DPPH* assay [11] with slight modifications. The same equipment as that described for the TPC and TAC was used. The extracts were dissolved in methanol to prepare a stock solution of 20 mg mL^−1^. This solution was further diluted to final concentrations of 15.6, 62.5, 125, 250 and 500 µg mL^−1^ before being transferred to a 96-well microtiter plate. Before adding DPPH*, each extraction solution was used as a blank. Each well contained 50 µL aliquot of the sample and 150 µL of DPPH* (400 µmol L^−1^). Each mixture was incubated at 37 °C for 30 min and then the absorbance at 517 nm was monitored. The absorbance value obtained was compared with that of the DPPH* solution measured immediately after being prepared, which was used as a reference. The absorbance decrease obtained indicated the percentage inhibition of the DPPH* by each dilution level of the samples. A plot of percentage inhibition versus concentration was made and the IC_50_ values were calculated using linear regression analysis. The experiments were performed in duplicate

### 2.7. Photochemiluminiscence (PCL) Activity

The antioxidant capacity was also determined using a PCL assay. This method was applied using a Photochem^®^ device (Analytik Jena AG, Jena, Germany) and it was conducted via an ACL protocol, which allows for the antioxidant capacity of the lipid-soluble components to be measured [12]. The study was performed using a commercial reagent kit ACL (Analytik Jena AG, Jena, Germany) [13]. For the assays, the extract was first dissolved in methanol:water (70:30) (about 30 g L^−1^). A 20 μL volume of this solution was then mixed with ACL reagent and the mixture was placed into the Photochem device. Results were calculated on the basis of standard curves into nanogram Trolox equivalents per microlitre of sample (ng µL^−1^).

### 2.8. Content of Phenolic Compounds

The content of individual phenolics was determined using HPLC. The equipment used for the measurements was an Alliance Separation Module 2695 chromatograph (Waters, Milford, CT, USA) with an automatic injector and a photodiode array detector 996 (DAD, Waters, Milford, CT, USA). The separation of the target compounds was accomplished on an ODS reverse phase (C_18_) column (250 mm × 4.6 mm i.d., particle size 5 µm, ACE, Madrid, Spain) at a flow rate of 1 mL min^−1^. To protect the column, an Altima 5 µm C_18_ pre-column (Altech, Barcelona, Spain) was used. Both the pre-column and column operated at 20 °C. The elution program was the same as previously optimised [14]. Blanks between consecutive runs were performed. Phenolic acids were registered at 320 nm, quercetin at 360 nm, quercertin-3-glucoside at 348 nm and cyanidin-3-*O*-glucoside at 520 nm. Stock solutions of the standard compounds were prepared in 70% (*v*/*v*) methanol to a final concentration of 1 mg mL^−1^. In addition, calibration curves of the standards were established for six data points and each standard dilution was run in triplicate. The extracts were also reconstituted in 700 mL L^−1^ methanol and also run three times at a concentration of 20 mg mL^−1^ for the quantification of polyphenols.

### 2.9. Statistical Analysis

An analysis of variance for the TPC, TAC, DPPH and PCL activities and individual phenolic content was carried out using the one-way analysis of variance (ANOVA) method. The results are presented as the average of all values obtained and standard deviation (mg kg^−1^ weight ± SD). Data from raw flour, heated flour and bread were included in the statistical analysis. Comparisons of means were made by using Fisher’s protected LSD. Differences were considered significant for *p* < 0.05.

## 3. Results and Discussion

### 3.1. Comparison between Millo corvo Corn Flour and White Corn Flour

Initially, raw *Millo corvo* corn flour was compared with conventional white corn flour in terms of the TPC and antioxidant properties, which were measured using two different assays (i.e., DPPH and PCL). PCL is a relatively new method for measuring antioxidant capacity. It is based on the optical excitation UV sensitiser that causes free radical generation, which is partially eliminated by naturally occurring antioxidants in the sample [15]. The results are summarised in Table 1. As seen, significant (*p* < 0.05) differences between the two flours were measured. *Millo corvo* corn flour exhibited significantly (*p* < 0.05) higher TPC and antioxidant activity from both the DPPH and PCL methods. Different TPC data were reported according to the corn cultivar [1,16]. However, all bibliographic studies on antioxidant activity agree on the higher antioxidant capacity of colored corn with respect to that of white ones [1] due to the higher content of natural pigments, mainly anthocyanins.

In a view of these results, the use of white corn flour to bake bread was discarded. The bread study was only carried out using *Millo Corvo* corn flour as an ingredient.

### 3.2. Effect of Temperature on the Antioxidant Content in Millo corvo Flour

The effect of temperature on TPC, TAC, DPPH scavenging activity and individual phenolics in raw *Millo corvo* flour was performed by reproducing the baking processing at 150, 180 and 200 °C.

As can be seen, the TPC values decreased significantly (*p* < 0.05) with heating. In particular, TPC dropped from 305.3 mg GAE 100 g^−1^ in the unheated flour samples to 119.6, 105.6 and 102.3 mg GAE 100 g^−1^ after heating at 150 °C, 180 °C and 200 °C, respectively. Interestingly, all the temperatures tested provided similar results.

Regarding TAC, values also diminished with heating. In particular, a significant (*p* > 0.05) decrease from 381 mg cyanidin-3-*O*-glucoside 100 g^−1^ in the unheated sample to 27 mg cyanidin-3-*O*-glucoside 100 g^−1^ after heating at 180 °C was measured. The apparent changes observed after heating at 150 °C and 200 °C were not considered since they were not statistically significant.

**Figure 1:**
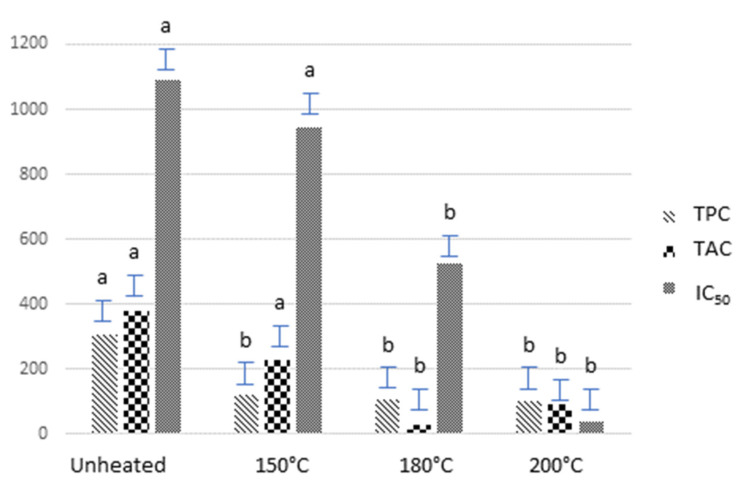
Depicts TPC (expressed as mg GAE 100 g^−1^), TAC (expressed as mg cyanidin-3-*O*-glucoside 100 g^−1^) and free radical scavenging activity (expressed as IC_50_, µg mL^−1^) measured in *Millo corvo* corn flour heated at different temperatures (i.e., 150, 180 and 200 °C) for 1 h. The results were compared with those of the unheated flour. Different letters (a, b) indicate differences at *p* < 0.05.

Concerning the DPPH scavenging activity, a similar trend was observed (see Figure 1). The IC_50_ value obtained for unheated flour (i.e., 1091.7 µg mL^−1^) was not significantly (*p* > 0.05) affected after heating at 150 °C (i.e., 943.9 µg mL^−1^). However, the application of 180 °C and 200 °C during the baking process did result in a significant (*p* < 0.05) increase in DPPH activity, particularly at 200 °C (i.e., IC_50_ value of 36.0 µg mL^−1^).

Table 2 summarises the contents of individual phenolics (expressed in g kg^−1^) in *Millo corvo* flour baked at different temperatures (i.e., 150, 180 and 200 °C) for 1 h. From Table 2, the level of all phenolics except *trans*-ferulic acid decreased significantly (*p* < 0.05) as a result of heating. This decrease suggests a direct correlation between the individual phenolics here determined and those contributing the most to the TPC (Figure 1).

As seen in Table 2, the decrease in the amount of anthocyanins was noticeable, particularly cyanidin-3-*O*-glucoside, which dropped from 0.48 g kg^−1^ in the control to 0.18 g kg^−1^ in samples heated at 150 °C. As compared with TAC (Figure 1), the heating effect was more noteworthy for individual anthocyanins than for TAC. In any case, the increase in temperature brought about the thermal degradation of anthocyanins in the studied conditions. As is also observed in Table 2, no significant (*p* > 0.05) differences were found among the temperatures tested. Hence, the individual phenolic content would be similar whatever the temperature applied.

There exist several bibliographic reports describing the direct relationship between the content of colored compounds, such as anthocyanins and carotenoids, and the antioxidant activity of a food [17,18]. Since the content of the major anthocyanin, namely, cyanidin-3-*O*-glucoside, decreased significantly with heating, the increase of the DPPH activity (Figure 1) was most likely due to Maillard reaction products being produced as a result of the high temperatures. In fact, the effect of Maillard reactions on the increase of the antioxidant activity has already been reported in cookies [19]. Accordingly, some non-enzymatic browning compounds are capable of stabilising anthocyanins via interaction with the resulting aglycone during the thermal degradation process. This is probably also the reason why TAC increased slightly at 200 °C (Figure 1). However, it is important to bear in mind that Maillard products are regarded as undesirable compounds since their formation involves the destruction of essential amino acids and the production of anti-nutritive compounds [20]. Therefore, baking temperatures higher than 150 °C are not recommendable. On the other hand, it is also worthy to point out that non-anthocyanin antioxidants, such as carotenoids (e.g., lutein and β-carotene) and certain non-colored compounds (e.g., ferulic acid), can also contribute to the antioxidant activity [21]. The use of extremely high temperatures might easily degrade these beneficial compounds. Overall, 150 °C was the selected temperature for the breadmaking process.

### 3.3. Comparison between Raw Millo corvo Flour and Millo corvo Bread

Table 3 represents the TPC and free radical scavenging activity of the homemade *Millo corvo* bread baked at 150 °C for 1 h. For comparison, the initial raw *Millo corvo* flour data are also included in Table 3.

From Table 3, TPC values decreased significantly (*p* < 0.05) from 291.1 mg GAE 100 g^−1^ in raw flour to 48.1 mg GAE 100 g^−1^ in bread. In contrast, TAC increased significantly (*p* < 0.05) from 381.2 mg g^−1^ in raw flour to 507.0 mg g^−1^ in bread, and the antioxidant activity in terms of DPPH was not significantly affected by heating, obtaining statistically insignificant values for raw flour and bread.

Although the effect of 150 °C on TPC and the DPPH activity in raw flour was similar in both cases, the results of TAC differed considerably (see also Figure 1). In fact, while baking at 150 °C did not significantly affect TAC in *Millo corvo* raw flour (Figure 1), it did result in a significant (*p* < 0.05) increase in *Millo corvo* bread as compared with the unheated raw flour (507.0 mg 100 g^−1^ vs. 381.2 mg 100 g^−1^, Table 3).

Table 4 presents the content of individual phenolics in homemade *Millo corvo* bread (expressed in g kg^−1^) baked at 150 °C for 1 h. In the same way, chlorogenic and caffeic acids, together with quercetin, kept decreasing after baking, whereas ferulic acid was not affected by heating. However, the breadmaking process did minimise the effect of the temperature on the contents of quercetin-3-glucoside and cyanidin-3-*O*-glucoside. As seen in Table 4, the content of both of them remained the same during the whole baking process. The higher cyanidin-3-*O*-glucoside content obtained in bread with respect to raw flour was in accordance with the higher TAC above mentioned (see Table 3). This indicates that the thermal degradation of anthocyanins was lower during the baking of the bread than during the heating of the raw flour. This was most likely due to the mildness of the temperature reached through the addition of water, whose efficiency was sufficient to avoid the thermal degradation of the most labile phenolics.

Finally, the statistical comparison between the free radical scavenging activity in terms of the DPPH of raw flour (i.e., IC_50_ value of 1022.2 µg mL^−1^) and the final bread (i.e., IC_50_ value of 875.3 µg mL^−1^, Table 3) indicates that the DPPH activity was not significantly affected either by the temperature or the presence of water. With a view to verifying this aspect, a PCL assay was also applied to the final *Millo corvo* bread. The value obtained was 916.19 ng eq Trolox µL^−1^, which was statistically similar to that of *Millo corvo* raw flour (i.e., 734.3 ng equivalents Trolox µL^−1^, Table 1). The PCL results support those of the DPPH assay. Therefore, the preservation of the antioxidant activity of the starting raw *Millo Corvo* flour in the final bread baked from it in the baking conditions applied here was confirmed.

To our knowledge, there is no bibliographic report on the antioxidant properties of *Millo corvo* corn bread. This is why our results cannot be contrasted with the literature. However, the influence of baking on anthocyanins in colored-grain wheat bread was recently studied [22,23]. These authors also conclude that baking does not significantly affect the anthocyanin content; hence, colored wheat genotypes are also adequate for bread production.

## 4. Conclusions

The present study demonstrated that *Millo corvo* corn bread possessed the same phenolic content and antioxidant activity as the starting flour as long as 150 °C was applied during the baking process. This indicates that *Millo corvo* is a suitable corn variety for bread production due to the remaining antioxidant content after baking. Our objective now is to extend these results to the study of other colored compounds and additional bakery products. In particular, the consideration of carotenoids and additional anthocyanins and the study of new parameters, such as the baking time and the addition of citric acid, are scheduled.

## Figures and Tables

**Table 1:** TPC (expressed as mg GAE 100 g^−1^) and antioxidant activity measured using DPPH (expressed as IC_50_, µg mL^−1^) and PCL (ng Trolox mL^−1^) methods in white corn flour and *Millo corvo* corn flour.

Sample	TPC	PCL	IC_50_
White corn flour	169.7 ± 0.12 ^a^	331.9 ± 0.09 ^a^	2182.2 ± 0.12 ^a^
*Millo corvo* corn flour	305.3 ± 0.15 ^b^	734.3 ± 0.15 ^b^	1091.7 ± 0.16 ^b^

Different letters in the same row indicate differences at *p* < 0.05 between baking temperatures. Experiments were carried out in triplicate.

**Table 2:** Content of individual phenolics in *Millo corvo* corn flour (expressed in g kg^−1^) at different heating temperatures.

Phenolics	*Millo corvo* Flour Samples			
	Unheated	150 °C	180 °C	200 °C
Chlorogenic acid	1.17 ± 0.09 ^a^	0.16 ± 0.05 ^b^	0.10 ± 0.03 ^b^	0.13 ± 0.06 ^b^
Caffeic acid	1.43 ± 0.08 ^a^	0.16 ± 0.06 ^b^	0.11 ± 0.06 ^b^	0.15 ± 0.03 ^b^
*Trans*-ferulic acid	0.05 ± 0.01 ^a^	0.03 ± 0.08 ^a^	0.05 ± 0.03 ^a^	0.13 ± 0.07 ^a^
Quercetin	1.77 ± 0.12 ^a^	0.18 ± 0.08 ^b^	0.07 ± 0.03 ^b^	0.16 ± 0.03 ^b^
Quercetin-3-glucoside	0.12 ± 0.04 ^a^	0.02 ± 0.05 ^b^	0.03 ± 0.02 ^b^	0.02 ± 0.02 ^b^
Cyanidin-3-*O*-glucoside	0.48 ± 0.03 ^a^	0.18 ± 0.01 ^b^	0.10 ± 0.01 ^b^	0.13 ± 0.02 ^b^

The values were estimated as mean ± SD (*n* = 3). Different letters in the same row indicate differences at *p* < 0.05 between baking temperatures.

**Table 3:** TPC (mg GAE 100 g^−1^), TAC (mg cyanidin-3-*O*-glucoside 100 g^−1^) and DPPH scavenging activity (expressed as IC_50_, µg mL^−1^) of *Millo corvo* corn flour mixed with water and homemade *Millo corvo* corn bread (expressed in g kg^−1^).

Sample	TPC	TAC	IC_50_
Unheated *Millo corvo* flour	291.1 ± 0.08 ^a^	381.2 ± 0.10 ^a^	1022.2 ± 0.18 ^a^
Homemade *Millo corvo* bread	48.1 ± 0.05 ^b^	507.0 ± 0.13 ^b^	875.3 ± 0.16 ^a^

The values were estimated as mean ± SD (*n* = 3). Different letters in the same row indicate differences at *p* < 0.05. Experiments were carried out in triplicate.

**Table 4:** Content of individual phenolics in unheated raw *Millo corvo* corn flour and homemade *Millo corvo* corn bread (expressed in g kg^−1^).

Phenolics	*Millo corvo* Flour Samples	
	Unheated *Millo corvo* Flour	Home-made *Millo corvo* Bread
Chlorogenic acid	1.17 ± 0.06 ^a^	0.08 ± 0.01 ^a^
Caffeic acid	1.43 ± 0.02 ^a^	0.08 ± 0.02 ^b^
*Trans*-ferulic acid	0.12 ± 0.01 ^a^	0.10 ± 0.02 ^a^
Quercetin	1.77 ± 0.03 ^a^	0.07 ± 0.01 ^b^
Quercetin-3-glucoside	0.09 ± 0.01 ^a^	0.08 ± 0.01 ^a^
Cyanidin-3-*O*-glucoside	0.48 ± 0.04 ^a^	0.32 ± 0.01 ^a^

The values were estimated as mean ± SD (*n* = 3). Different letters in the same row indicate differences at *p* < 0.05 between baking temperatures.
